# A Malignant Squeeze: A Rare Cause of Cardiac Tamponade

**DOI:** 10.1155/2018/5470981

**Published:** 2018-09-23

**Authors:** Elisa Quiroz, Adam Hafeez, Ramy Mando, Zhou Yu, Feroze Momin

**Affiliations:** ^1^Department of Internal Medicine, Beaumont Health, Royal Oak, MI, USA; ^2^Department of Hematology and Oncology, Beaumont Health, Royal Oak, MI, USA

## Abstract

Primary cardiac lymphoma (PCL) is a rare condition described as a lymphoma localized to the heart or pericardium. Although cardiac involvement is seen in 10–20% of non-Hodgkin's lymphomas, PCL is extremely rare. It comprises merely 0.5% of all lymphomas and 1.3–2% of cardiac malignancies. Early detection is essential to avoid potentially fatal complications, and prognosis is highly dependent on the management of cardiac complications. The etiology of PCL is still unknown, and molecular characterization has yet to be studied leaving a great deal of research to be done in order to gain a better understanding of this rare disease process. We discuss the case of an 85-year-old female presenting with dyspnea and chest pain. Computed tomography of the chest revealed a pericardial effusion, and subsequent echocardiogram demonstrated a large circumferential effusion. She underwent emergent pericardiocentesis. Morphologic and immunophenotypic features were consistent with high-grade B-cell lymphoma with *t*(8; 14), and the patient was started on rituximab, cyclophosphamide, doxorubicin, vincristine, and prednisone (R-CHOP) with excellent response.

## 1. Introduction

Primary cardiac lymphoma (PCL) is a rare condition that is described as a lymphoma localized to the heart or pericardium with no extracardiac involvement. Although cardiac involvement is seen in about 10–20% of non-Hodgkin's lymphomas, PCL is extremely rare. It comprises merely 0.5% of all lymphomas and 1.3–2% of cardiac malignancies [1, 2]. Clinical presentation typically correlates with the region of the heart involved. It is often a clinical emergency as these patients commonly present with heart failure, cardiac tamponade, or arrhythmia [3]. Herein, we describe a rare case of PCL causing cardiac tamponade in an elderly patient.

## 2. Case Description

**Figure 1:**
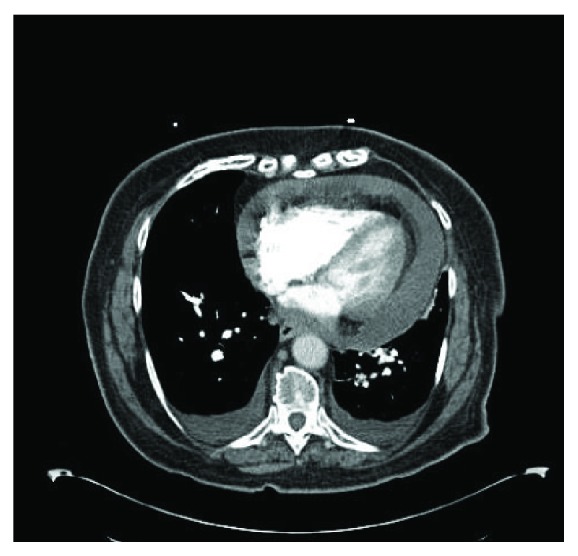
Computed tomography of the chest demonstrating pericardial effusion.

**Figure 2:**
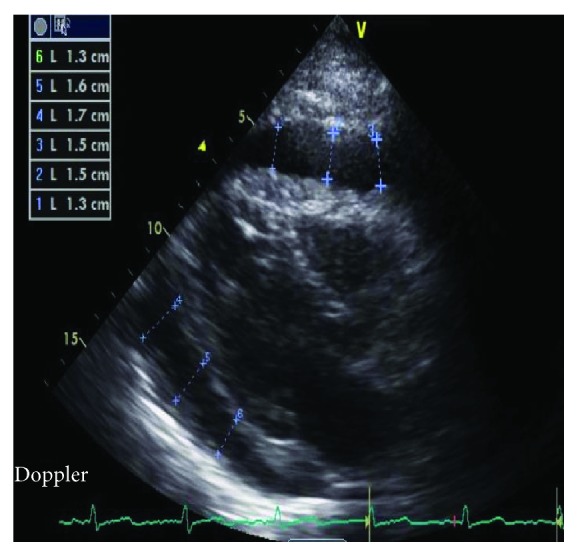
Echocardiogram demonstrating a large circumferential effusion (outlined) concerning for impending tamponade.

**Figure 3:**
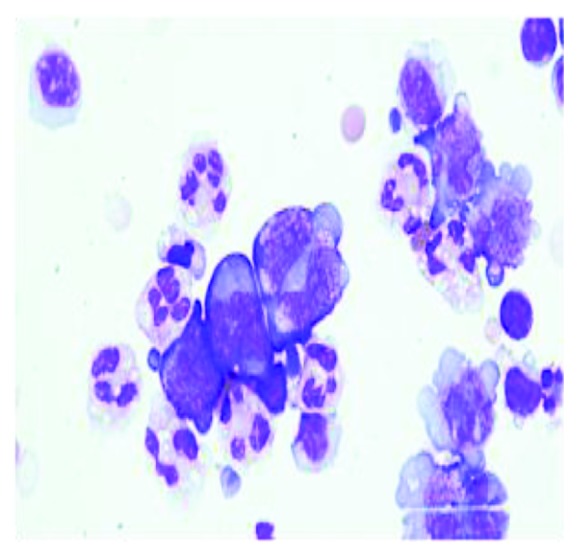
Histopathology remarkable for monoclonal B-cells with significant large cell components.

**Figure 4:**
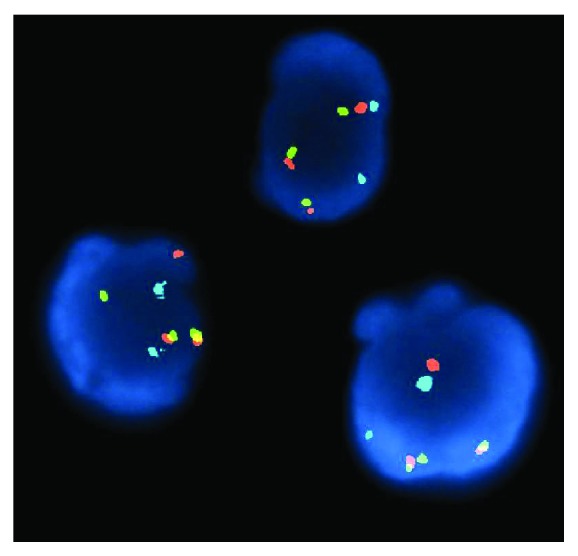
Fluorescence in situ hybridization demonstrating *t*(8; 14). This translocation is between IGH on chromasome 8 and MYC on chromosome 14.

An 85-year-old female with past medical history of recurrent deep venous thrombosis, pulmonary embolism on anticoagulation with a vena cava filter in place, rheumatoid arthritis, hypertension, hyperlipidemia, hypothyroidism, and type 2 diabetes mellitus presented to the emergency department with complaint of exertional dyspnea and chest pain. She denied fever, chills, or lower extremity edema and had no history of malignancy, weight loss, or night sweats. Initial vitals revealed BP of 86/62 mmHg that decreased to 79/60 mmHg with inspiration. Initial pulse was 95 bpm, and respiratory rate was 20. White blood cell count was mildly elevated at 11.5, troponins were normal, and electrocardiogram was unremarkable. D-dimer was elevated at 1290. Patient was sent for CT scan to evaluate for pulmonary embolism, and a moderate pericardial effusion was found (Figure 1). Subsequently, an echocardiogram was done to further delineate the effusion which revealed a large circumferential effusion with mild respiratory variation concerning for impending cardiac tamponade (Figure 2). She was admitted to the intensive care unit and underwent emergent pericardiocentesis. Flow cytometry of the pericardial fluid revealed a population of monoclonal B-cells with significant large cell component (Figure 3). The overall morphologic and immunophenotypic features were consistent with high-grade B-cell lymphoma with *t*(8; 14) (Figure 4). Bone marrow biopsy demonstrated monotypic B-cells compatible with the diagnosis of large B-cell lymphoma. The patient was started on rituximab, cyclophosphamide, doxorubicin, vincristine, and prednisone (R-CHOP) with an excellent initial response. She was transferred out of the ICU within days and discharged home for outpatient follow-up.

## 3. Discussion

**Figure 5:**
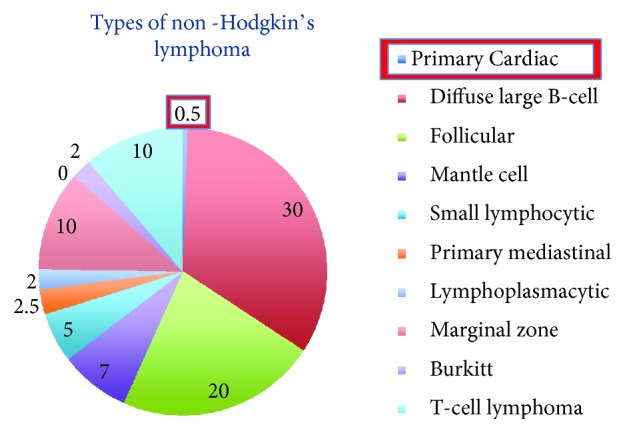
Graphical representation of frequencies of subtypes of non-Hodgkin's lymphoma.

Primary cardiac tumors are known classically to be rare and benign. Cardiac myxoma is the most frequently reported primary cardiac tumor, but hematologic disease, in rare cases, has been known to orginate in the heart [4]. Lymphomas compromise only 0.5–2% of primary cardiac tumors and occur most often in immunocompromised patients, such as those with human immunodeficiency virus (HIV) [5]. The frequencies of other types of non-Hodgkin's lymphoma are detailed in Figure 5 with diffuse large B-cell lymphoma comprising the majority at 30% [6]. PCL does occur in immunocompetent hosts although infrequently, as it did in our patient. In these immunocompetent patients, studies have shown that 80% of PCL are of diffuse B-cell lymphoma [7]. The location of cardiac involvement for PCL is often the right atrium and right ventricle. Clinical presentation can vary depending on the site of involvement of the heart. Most often, an extensive multidisciplinary workup is employed to come up with the correct diagnosis. Imaging modalities such as transthoracic and transesophageal echocardiography, computed tomography (as done in our patient), and magnetic resonance imaging are all utilized in diagnosing this entity. Confirmation of the diagnosis is through cytology of biopsy or pericardial fluid. The combination of chemotherapy and radiation is the treatment of choice. Treatment for B-cell lymphoma is typically an anthracycline-based, such as given in our patient. Our patient was treated with rituximab, cyclophosphamide, doxorubicin, vincristine, and prednisone (R-CHOP). Survival has been demonstrated in some cases up to 5 years with treatment but is less than a month without it [7].

Although PCL is rare, the pathophysiology is classic and detection through a detailed physical exam and directed workup diagnosis can be promptly made, even when presenting as tamponade as did our patient. Patients presenting with signs and symptoms of new-onset acute decompensated heart failure can potentially have this diagnosis. Immediate detection is essential to avoid fatal complications, which include cardiac tamponade, ischemia, and fatal arrhythmia. To our knowledge, only 13 cases have described PCL presenting with tamponade physiology and are highlighted in Table 1. Parato et al. describe a case of a younger patient who presented with cardiac tamponade due to PCL. The patient was treated with management of cardiac tamponade, surgical excision of the mass, and chemotherapy. Complete remission was obtained after six months of chemotherapy [8]. Although there was a large age difference, our patient also responded to chemotherapy following medical stabilization. Interestingly, Frikha et al. describe a case of an elderly immunocompromised patient presenting with cardiac tamponade along with paroxysmal third-degree atrioventricular (AV) block. They hypothesized that electrical compromise occurred via extension of the malignancy into the interatrial septum and the nodal tissue [9]. Similarly, Gowda and Khan and Houchaymi et al. describe a case of an elderly immunocompetent male who was found to have pericardial tamponade along with third-degree AV block. However, pericardial fluid analysis did not reveal the diagnosis until a transvenous biopsy of the cardiac tumor revealed non-Hodgkin large B-cell lymphoma [7, 10]. Gowda and Khan and Chiba et al. report a similar case of a middle-aged male with cardiac tamponade and AV block who was found on right atrial tumor biopsy to have a diffuse large B-cell lymphoma. The patient underwent chemotherapy and required permanent pacemaker placement [7, 11]. On our extensive literature search, we identified one case report in which the cardiac tamponade was not caused by the lymphoma itself, but rather due to the malignant right atrial rupturing causing tamponade [12]. Further cases are described in Table 1.

The etiology of PCL is still unknown, and molecular characterization has yet to be studied leaving a great deal of research to be done to gain a better understanding of this rare disease process. Nevertheless, although extremely rare, PCL can present with severe cardiac dysfunction due to tamponade physiology or impending tamponade physiology and heart failure. Clinical suspicion must remain high, and diagnostic studies of pericardial fluid can help prevent delay in management of an aggressive malignancy.

## Figures and Tables

**Table 1:** Reports of primary cardiac lymphoma causing cardiac tamponade.

Article (language, PMID)	Description	Highlights
Parato VM, Muscente F, Scarano M. Primary cardiac lymphoma: a case report. G Ital Cardiol (Rome). 2017 Jan; 18(1): 11–13 (Italian, 28287210)	35-year-old immunocompromised patient presented with signs and symptoms of tamponade. Echocardiography revealed lateral AV mass with large pericardial effusion. Patient had surgical excision of mass followed by chemotherapy that induced complete remission at 6 months.	
Tzachanis D, Dewar R, Luptakova K, Chang JD, et al. Primary cardiac Burkitt lymphoma presenting with abdominal pain (English, 25431699)	44-year-old female presented with abdominal bloating who was found to have a large pericardial effusion with tamponade physiology. Pericardial fluid studies led to diagnosis of cardiac Burkitt lymphoma.	Primary Burkitt lymphoma
Frikha Z, Abid L, Abid D, Mallek S, et al. Cardiac tamponade and paroxysmal third-degree atrioventricular block revealing a primary cardiac non-Hodgkin large B-cell lymphoma of the right ventricle: a case report. J Med Case Rep. 2011 Sep 5; 5: 433 (English, 21892927)	64-year-old immunocompromised male presented with cardiac tamponade as well as paroxysmal third-degree AV block. He was found to have a large RV mass. Following excision, histology confirmed non-Hodgkin large B-cell lymphoma.	Primary non-Hodgkin large B-cell lymphoma 3rd-degree AV block
Houchaymi Z, Helou S, Ballout J. Pericardial tamponade and third-degree atrioventricular block revealing a primary cardiac lymphoma. Rev Med Interne. 2010 Nov; 31 (11): e4-6 (French, 20605278)	78-year-old immunocompetent male presented with pericardial tamponade and third-degree AV block. Biopsy of the cardiac tumor showed non-Hodgkin large B-Cell lymphoma.	Primary non-Hodgkin large B-cell lymphoma 3rd-degree AV block
Chiba Y, Oka K, Saito H, et al. Primary cardiac B-cell lymphoma presented as heart tamponade and atrioventricular block: a case report. Acta Cytol. 2010 Jan–Feb; 54 (1): 79–81 (English, 20306995)	49-year-old male with cardiac tamponade and AV block. Pericardial effusion and transvenous biopsy confirmed diagnosis of diffuse large B-cell lymphoma. He underwent chemotherapy and permanent pacemaker placement.	Diffuse large B-cell lymphoma AV block
Legault S, Couture C, Bourgault C, et al. Primary cardiac Burkitt-like lymphoma of the right atrium	74-year-old male with dyspnea found to have a large pericardial effusion with tamponade physiology. Found on pericardial fluid studies and biopsy to have Burkitt-like PCL.	Burkitt-like PCL
Ling LF, Chai P, Kee AC, et al. Primary cardiac lymphoma presenting with cardiac tamponade. Am Heart Hosp J. 2009 Winter; 7 (2): E125-7 (English, 19279985)	55-year-old immunocompetent man presented with pyrexia initially and was found later to have cardiac tamponade. He was diagnosed with PCL.	
Gosev I, Siric F, Gasparovic H, et al. Surgical treatment of a primary cardiac lymphoma presenting with tamponade physiology (English, 16846425)	67-year-old immunocompetent presented with dyspnea. Found to have tamponade physiology on echocardiography. Surgical biopsy confirmed diffuse large B-cell lymphoma of centroblastic subtype.	Diffuse large B-cell lymphoma of centroblastic subtype Surgical diagnosis, no pericardiocentesis performed
Wilhite DB, Quigley RL. Occult cardiac lymphoma presenting with cardiac tamponade. Tex Heart Inst. J. 2003; 30 (1): 62–4 (English, 12638674)	83-year-old Filipino male with dyspnea found on echocardiogram to have a large, homogenous pericardial effusion with RA collapse. He was treated with subxiphoid pericardiostomy and had the pericardial drain removed the next day as he clinically improved. He returned two weeks later with same symptoms and again found to have reaccumulating pericardial fluid requiring urgent anterior pericardiectomy.	Failure of traditional pericardiostomy
Menotti A, Imperadore F, Pelosi G. et al. Heart rupture at the right atrial level as the first manifestation of malignant lymphoma. Cardiologia. 1996 Jan;41(1):65–7 (Italian, 8697472)	Malignant lymphoma presenting as cardiac tamponade due to right atrial rupture.	Right atrial rupture causing the tamponade
Roller MB, Manoharan A, Lvoff R. Primary cardiac lymphoma. Acta Haematol. 1991; 85 (1): 47–48 (English, 2011932)	Elderly male with cardiac tamponade found on pericardial fluid analysis to have PCL.	
Nagamine K, Noda H. Two cases cardiac lymphoma presenting with pericardial effusion and tamponade. Jpn Circ. J. 1990 Sep; 54 (9): 1158–64 (English, 2266577)	Two cases of males in their 70s presenting with dyspnea who were found to have pericardial effusion with tamponade physiology on echocardiogram.	
Pozniak AL, Thomas RD, Hobbs CB, et al. Primary malignant lymphoma of the heart. Antemorten cytologic diagnosis. Acta Cytol. 1986 Nov–Dec; 30 (6): 662–4. (English, 3466501)	Patient who presented with ventricular arrhythmias and was found to have pericardial effusion with impairment of LV contraction. Postmortem examination showed malignant lymphoma confined to myocardium.	
Patel J, Melly L, Sheppard MN. Primary cardiac lymphoma: B- and T-cell cases at a specialist UK centre	Case series of six patients presenting with wide spectrum of symptoms. Most cases involved 2 or more chambers of the heart. Presentations included conduction disturbances, effusion, valvular dysfunction, heart failure, stroke, and sudden death.	Clinical presentation of PCL (B- and T-cells)
